# Rethinking obstetric violence and the “neglect of neglect”: the silence of a labour ward milieu in a South African district hospital

**DOI:** 10.1186/s12914-019-0218-2

**Published:** 2019-10-30

**Authors:** Maura Lappeman, Leslie Swartz

**Affiliations:** Department of Psychology, Stellenbosch University, Private Bag X1, Matieland, 7602 South Africa

**Keywords:** South Africa, Obstetric violence, Milieu, Neglect, Qualitative

## Abstract

**Background:**

Research into the mistreatment of women during childbirth has increased over recent years. Overt violence is an important focus of research, but recently there has been increasing recognition that there are other ways in which women in labour may be uncared for or even hurt. As part of a larger study focussing on staff responses to stillbirths, we wanted to gain contextual information on how high risk pregnancies are handled in general in Khayelitsha Hospital, a district hospital in an impoverished urban setting in the Western Cape Province of South Africa. This health care system experiences an immense patient load, the poverty of the community it serves, and the numerous traumas affecting both patients and staff.

**Methods:**

In order to obtain rich exploratory data, a qualitative research methodology was used. The primary data source was observations in the labour ward, interviewing labour ward staff (doctors, nurse, and cleaners). The secondary data source was the analysis of hospital documents, specifically those related to labour ward policy.

**Results:**

From our numerous observations and discussions, it is clear that no one is being overtly mistreated in this hospital and patients are medically well attended to. Although we saw no physical abuse, we noted the silence in the ward. Beside medical related interactions, we also noted that there were limited interactions between the women and the health care providers.

**Conclusions:**

Silence can be a form of neglect as it leaves the women feeling uncared for and not seen. In an overburdened health care system where both staff and patients are often overwhelmed or traumatised, silence can be a way in which a system defends itself against what it knows it cannot provide.

## Background

In recent years there has been growing interest in the mistreatment of women in childbirth [1–11]. The term “obstetric violence” originated in South America in 2007, and is often used for this particular kind of maltreatment [2, 9]. Chadwick [5] specifically defined the concept of obstetric violence as the disrespectful, aggressive and humiliating treatment of women and girls during labour and birth. Morales et al. [2] further argue that obstetric violence is an expression of violence during the provision of health care, which occurs in a social environment favouring the development of power relationships between patients and health care practitioners. Its origin may lie in a health care system where political and economic foundations encourage inequality based on the patients’ buying power [2]. In its entirety, the term encompasses problematic practices such as neglect, verbal and emotional abuse, physical abuse, and sexual abuse, lack of confidentiality and consensual care, and inappropriate use of medical intervention, such as episiotomies, inductions and unnecessary caesarean sections.

When reviewing the literature on childbirth in South Africa and a number of other countries, themes that emerge are overt aggression, lack of support, lack of privacy and gender inequality. Indeed, even in South Africa, a large divide exists between those able to access private healthcare and those forced by poverty to rely solely on the services offered by the government. Lambert et al. [7], however, contextualised some of the challenges with simplistic approaches to childbirth by expressing that “an approach that ignores the relationships and culture central to care provision is fundamentally flawed” (p. 256). A growing body of current literature is highly critical of medicalised and overtly violent obstetric environments. When specifically researching obstetric violence in South Africa, Chadwick (p. 423) [5] reported that:

*“Violence in obstetric contexts in SA is multi-layered and complicated by the fact that it includes both individual acts of abuse and structural components such as degrading spatial configurations that lead to lack of privacy and impede the use of labour companions.”*

Within the boundaries of Chadwick’s [5] conclusion is the notion that abusive structural contexts may even include good medical care. The concept of obstetric violence is helpful and provocative, as it focusses attention on the nature of abuse of women during labour – a very important public health problem. But although overt violence is an important focus of research, recently there has been increasing recognition that there are other ways in which people may be uncared for, or even hurt. Nixon [12], for example, speaks of what he terms “slow violence”, which he defines as “a violence that occurs gradually and out of sight; a delayed destruction often dispersed across time and space” (p. 2) [12]. Chadwick [4] also wrote on a similar violence which she named “gentle violence” where women become submissive, compliant body subjects who willingly accept their role as patient. In the context of health care (including obstetric care), even where there is no overt violence or ill-will toward women giving birth, there are ways in which care practices, however well-meant, may not be best for these women and may even have deleterious consequences. This brings to light the idea that obstetric violence can be the result of structural design and well-intentioned care.

In this study we specifically look at a labour ward milieu in a government hospital in South Africa. This study was part of a larger study focussing on staff responses to stillbirths, and resulted from our need to gain contextual information on how high risk pregnancies are managed in general in the hospital.

### Understanding institutional care

Isabel Menzies Lyth [13] was instrumental in foundational theory on creating better hospital environments. While not specifically focused on the labour ward, Lyth researched nursing services in a general hospital in Britain in the 1950s. Her research is considered a valuable tool for understanding the dynamics of relationships in hospitals. She noted that health care providers developed practical strategies for mediating their inherently stressful contact with patients [14]. Lyth addressed what today is termed “structural violence”, which is the unseen expression of violence weaved into the fabric of society, perpetuating social inequalities [15–18].

Milieu may form a component of structural violence in medical contexts, and has primarily been studied in the context of inpatient psychiatric holding environments [19]. Milieu [20] is defined as the physical or social setting in which something occurs or develops. Milieu also includes the feeling or mood associated with a particular place, person, or thing [20], and may affect clinical outcomes [19, 21]. In a hospital context, patient-practitioner interactions are crucial for creating the ward milieu. These hospital ward interactions act as a “social defence system (which) develops over time as the result of collusive interaction and agreement, often unconscious, between members of the organisation as to what form it will take” (p. 11) [13]. Since obstetric violence is a manifestation of structural violence, the two phenomena share many of the same features. Chadwick [5] does not specifically look at milieu, but the results of Lyth’s [13] research strongly suggest that the milieu of a labour ward may increase or decrease the potential for obstetric violence.

Informed by the work of Menzies Lyth, and as part of a larger study on obstetric care practices, we report here on a context in which it would be incorrect to speak about obstetric violence. However, we believe it is important to think about structural arrangements which are not optimal for women. We are interested in what occurs where no one is being mistreated and birthing mothers are medically well attended to. We show in this article how in a hospital in a stressed public health system, we found disengagement, alienation and depersonalisation of both the birthing mother and the health care practitioners. As we shall show in the results section, the trauma was found not in overt violence or acts of perpetration, but in the silence of a ward where no one was talking to anyone and all were left unseen. How do we understand this form of invalidation? In raising this question, not about abuse but about more subtle (and possibly more pervasive) forms of invalidation, we are reminded of the concept introduced by Music [22], in a very different context, of “neglecting neglect”. Music [22] notes that in studies of childcare practices, though it is essential, for good reasons, to focus on abusive practices, what is often less considered is the ways in which neglectful practices (not always overtly ill intentioned) may have deleterious effects. We shall show in this paper how good quality care from a technical point of view may be compromised by silence and invalidation – by what may be termed a form of defensive neglect.

### Location of the study: Khayelitsha hospital, Cape Town, South Africa

The research took place in Khayelitsha Hospital (KH). KH is a large district hospital with 340 beds situated in Khayelitsha, the second biggest Black township in South Africa after Soweto in Johannesburg [23]. South African townships developed as a result of the Apartheid regime’s attempt to enforce the Group Areas Act of 1950. This act, which designated separate residential areas along racial lines, forcing people of colour to live in areas further away from the city centre, often in dire living standards [24]. South Africa has a growing population. Midyear of 2018 the population of South Africa was 57, 73 million people [25]. The *Recorded live births, 2017* report released by Statistics South Africa today shows that a total of 989,318 births were registered in South Africa in 2017. In the last 10 years the population of Khayelitsha has risen from 400,000 to 2.4 million, 50% of whom are under 19 years of age [26]. The area’s unemployment rate is 73%, with 70% living in temporary structures called shacks [26]. Eighty-nine percent of homes are considered moderately to severely food insecure. The extreme poverty, and poor community infrastructure, has led to surging crime rates, gangs, violence, drugs, as well as other societal ills [27]. Khayelitsha is currently known as the murder capital of South Africa, with attempted murder increasing by close to 30% in 2012/13 [26].

KH opened in 2012 and is the only district hospital serving the Khayelitsha area. The hospital is well resourced with medical doctors and some of the latest medical treatment facilities in the Western Cape [28]. There is a 24-h theatre facility availability, and a CT scan and ultrasound department. There is also a women’s health unit. In the obstetric team, there are two full time qualified obstetricians with many years of experience. There are also four obstetric registrars rotating through the hospital as the hospital is linked to a tertiary education centre. With regards to obstetrics, only women with high risk pregnancies are brought to the hospital to deliver, otherwise they would deliver at the community health clinic’s maternity units. With this in mind, higher anxiety would be expected amongst the staff and patients than might be expected in a community clinic.

In an organisation like KH, the health care system experiences an immense patient load, the poverty of the community it serves, and the numerous traumas affecting both patients and staff. During staff interviews, the hospital healthcare workers from the Khayelitsha community expressed exposure to rape, neglect, financial struggles, domestic violence, murder of loved ones and their own obstetric trauma. These challenges are overlaid on an apartheid history, and on-going cultural and language differences between patients and staff, and within the staff group itself. Specifically, women in the Khayelitsha community are often devalued [29, 30], and teenage and unwanted pregnancies are common, which lead to poor pre-natal care, often because of shame or lack of knowledge [31].

## Methods

In order to obtain rich exploratory data, a qualitative research methodology is regularly used. Ethnographic research is known to produce a trustworthy and descriptive qualitative accounts of complex phenomena [32]. In addition, using multiple sources of data helped us to triangulate the findings.

### Ethnographic sampling and data collection

This study employed an ethnographic research model, which entails trying to understand practices in a particular setting [32–34]. The data comprised three sources of primary data and one source of secondary data. The primary data source was observations in the labour ward, interviewing labour ward staff (doctors, nurse, and cleaners) and patients who used the ward. The secondary data source was the analysis of hospital documents, specifically those related to labour ward policy.

#### Participant observation

Employed as a Clinical Psychologist at KH since 2014, the first author (we use the pronoun “I” for the first author as data collector throughout) was a participant observer, and this was the primary source of ethnographic data. I had the dual role of maintaining the stance of an observer while developing an understanding of how health care practitioners and patients experience the labour ward at KH. As suggested by Hollway et al. [35], I relied heavily on supervision in this complex task. Psychoanalytically informed research relies on our minds to aid reflection on the data. A research journal was used to record observations of the day-to-day practices in the labour and antenatal wards. This journal included descriptions of the difficulties – both practical and ethical – which I encountered during the process. It described my subjective and internal ruminations regarding the ward and individual participants – both patients and health care practitioners – as well as considerations of my own engagement with the data and procedures. The journal provided me with important supplementary data in terms of insights into my own processes which may have been lost without a written record. The journal informed and promoted the reflexivity which is acknowledged to be an important component of responsible qualitative research [35, 36]. In the 6 week observation period, I also reviewed my clinical notes on previous patients that I had consulted in the labour ward. Throughout the observation period, I reviewed my notes by identifying and developing themes.

Every day I would spend at least an hour just observing the daily rituals in the ward over a period of 6 weeks, although I am familiar with the ward, having been working at the hospital for 5 years. My presence was duly noted and at times caused anxiety. A cleaning staff member thought I was from Cutting Edge (an investigative television show exposing shameful practices), whereas others thought I was monitoring their behaviours to criticize them at their next performance review. In this study we have chosen a psychoanalytic paradigm which assumes that anxiety is inherent in the human condition.

Psychoanalysis, particularly the work of Melanie Klein, has contributed significantly to our understanding of the nature of anxiety and the primitive defences. These defences enable us as individuals to defend against the anxiety, which otherwise may overwhelm and destroy. If anxiety is the basis of the way in which the individual operates, then the protective defence of anxiety is essential for the stability of the individual and the individual’s ability to engage in a threatening world.

#### In-depth staff interviews

Sixteen labour ward staff interviews were conducted at KH, which included obstetric consultants, medical doctors, nurses and a clinical psychologist. Purposive sampling was used. Each interview was about an hour. Hollway and Jefferson [37] postulate that interviewees are psychically defended, which implies that everyone has an unconscious which contains motivations, instincts and impulses which are constrained by the social and political world in which they live. A defended subject may not tell a complete and transparent story, this being either a conscious or unconscious act. Some topics or memories are too difficult to talk about because they threaten to break down emotional defences. This may be seen to be true in KH where there is much trauma.

To ensure practicality, the interviewing process begins with a free association narrative interview technique (FANI) in which the questioning style is kept as open as possible, letting the interviewee’s ideas, views and story emerge as much as possible in their own words. This allows the interviewer to look critically at any inconsistencies and contradictions. The interviewing process is then followed by a detailed analysis of the experience as a whole, including the relationship between the interviewer and interviewee, the emotions involved, examining the words in the transcript, and a careful consideration of the narrative construction. We did also interview patients, but this article focusses on staff experiences and understandings, so these data will be reported elsewhere.

#### Document and policy analysis

Hospital documents, official policies and any protocols relating to the labour ward and care of mothers were also collected for analysis. In particular, the monthly maternity report from January to May 2018 was analysed, as well as doctors’ and nurses’ clinical notes Table 1.

**Table 1:** Summary of the research methods used in this study

Research Participation	Research Method	Data Collection	Data Capturing	Data Management	Data Analysis
Labour ward observation at KDH	Ethnography	Own process notes Observation Hospital documents	Field journal	Interpretation of field notes Triangulate with data from B and C	Observational data examined and interpreted for common themes
Staff in labour ward: • Doctors • Nurses • Community service Psychologist	Free Association narrative Interviews	Free Association narrative Interviews	Digital voice recorder Research Journal	Transcription Triangulate with data from A and C	Analysis of content further using FANI methods.

The data collection took a total of 21 weeks. The first 6 weeks were purely observational, and the interviews were conducted in the remaining weeks. Informal briefing sessions were conducted a week prior to the formal interviews so that interviewees could ask questions and decide whether or not to participate. As I work at the hospital full time, the medical doctors and nurses who participated in the study asked me about themes that were emerging from the data. They gave me feedback which helped in verifying the interpretations of themes as they emerged from the data, as well as the validity of subsequent results. The participants were able to add information to confirm aspects of data, or ask that inaccurate interpretations be removed. The audio recordings, transcripts and notes from the observations and interviews were kept in my office at work under password protection, and were only read by my academic supervisor and myself.

### Ethical considerations

Ethical clearance was obtained from the Humanities Research Ethics Committee at Stellenbosch University (REC-2018-1844) and the Western Cape Department of Health (WC 201801 033) before proceeding with my research and data collection.

Participation in the study was voluntary. An information leaflet was included in the informed consent form, and written consent was given by all participants. The information leaflet explained the purpose and objectives of the study, what participation entailed, the possibility of harm or risk, as well as the potential benefits of the study. The leaflet also outlined how confidentiality and anonymity would be maintained, and the participants’ right to withdraw from the study. Contact information for all those involved in the study were also provided, including my supervisor and the details of the contact person at the Division for Research Development of Stellenbosch University, should they have any further questions. No payment was offered for participation. I did, however, provide refreshments at my own cost. Confidentiality was assured by the use of pseudonyms. I explained to each participant that they were able to leave the study at any point and their story would not be included in the research. No participant withdrew from the study.

An option of counselling was offered to the participants. None of the staff requested further counselling, however if they had, they would have been referred to ICAS which is a governmental health and wellness programme which offers employees counselling.

For ethnographic observation in the labour ward, an explanation sheet of the research was put up in the ward before I started with observations. I spoke regularly to the staff about my research at the ward round and gave a space for staff to ask any questions or make any objections. I attempted to remain unobtrusive and did not initiate direct interaction with ward staff or patients during data collection. I remained respectful of relationships between staff and patients, did not impinge on the ward environment, and tried not to cause any upset to any person during the observations.

### Data analysis

The analysis of the data utilised the guidelines provided by the principles of FANI by Wendy Hollway and Tony Jefferson [32]. The FANI method pays close attention to the emotions, thoughts, and motivations of the interviewee, as well as taking into account unconscious dynamics and processes. The relationship between the interviewer and interviewee is seen as crucial as both come to the interview situation with their own anxieties, defences, political and social positioning and histories, which can affect the data that are created.

The combination of ethnographic data, the hospital documents and the interviews provided a deep, rich understanding of the emotional experiences of staff and patients in a highly stressed health care system. The analysis focussed on the most relevant emerging themes as well as on a deepening understanding of what was happening around me consciously and unconsciously, as far as possible. Scripts and the journal were read and re-read and reflected upon.

## Findings

During the research, a set of themes emerged that directly contributed to the labour ward milieu. We discuss themes under two main headings: The world of the hospital and the world the mother brings. Contributions came from the hospital structure and polity itself as well as staff and patients. We discuss the details of these findings below and the significance of the findings are further discussed thereafter.

### The world of the hospital

Walking into the ward, the first encounter is with the security guard who asks any visitor what their purpose is and writes down the particulars of the person. Then one would walk down the passage, passing the doctors’ office and the nurses’ tea room. The doctors’ office has computers, a coffee machine and fridge. It is often filled with various foods such as fruit and muffins. The nurses’ tea room is equipped with a fridge, lockers and a large table where the nurses/cleaners can bring their own lunch. I thought about the separate tea rooms as indicative of a social environment favouring the development of power relationships between the medical doctors and nurses; and wondered how this plays out with the staff and the patients. Evidence for this reality came from one of the interview participants who stated,

*“Because sometimes there might be a misunderstanding between the nurse and the doctor. Because sometimes you figure out there is a problem, but the problem now, you will tell the doctor, and then the doctor doesn’t act the way that they’re supposed to act. And you feel like if you were in that authority you are going to do something for this patient, but at the same time you are not the one who is doing the decision because at the end it’s going to depend on the doctor for that decision. So when things go wrong you will blame yourself.” (Professional Nurse & Midwife M, 35 years old)*

*“If the doctor is not doing anything, you cannot just decide to take the patient to theatre yourself. So that’s another thing. Otherwise, with the other things, there are some doctors who will understand, especially the permanent ones, we understand each other. But the problem is the locum one, the ones who are working in some places, you are just a nurse and you cannot tell me what to do.” (Professional Nurse & Midwife M, 35 years old)*

There is a nursing station in the middle of the ward. It is mainly functional. No staff greets incoming patients or other staff. The staff will engage in conversation if individually addressed. No hostility is noted in their tone.

The ward has an admission room, an induction room, a postnatal room, five delivery rooms with sliding doors and a quiet room allocated for women who have had an IUD or miscarriage. The ward is well maintained and clean. The admission room, induction room and postnatal room are able to accommodate four women each. Curtains are closed around a mother when the staff is assessing or examining her. For this reason, women in these rooms are not allowed to have visitors because of the privacy of the other patients. During active labour, women are brought to the delivery room and the door is shut for privacy. The women generally deliver on the bed although there are large birthing balls. Since the women are alone in the delivery room, visitors are allowed, although few come, as discussed below. Occasionally there are brief grunts and muffled screams with the surges of labour pains, but then the ward returns to a silent milieu.

The consultant asked me to make special mention about the windows of the delivery rooms that look onto the gardens. Reportedly, workers in the gardens often look through the windows whilst a woman is in labour until they are shooed away by the doctors or the nurses. He laughed telling me, but again I thought about the physical design and why no one thought about putting in one way glass so the mothers can look outside but no one can look in.

#### Long periods of silence

During my daily observations, I would sit quietly and wait. The ward was quiet around me (even the new-born babies barely cry). Only the occasional nurses speak to each other in Xhosa at the nursing station. A clinical psychologist noted,

*“It’s quiet. Because the noisier one is the postnatal. But I mean, even when you walk into the labour ward, it’s warm, temperature-wise, it’s very warm. I remember, last week it was cold in the passage, but as soon as I walked in there, nice and warm. But it’s so quiet. Because now I’m thinking this quiet is almost like a … it’s almost weird and cold.” (Community Service Clinical Psychologist, 33 years old)*

The contrast of a typical bustling labour ward stereotype and this reality was significant.

#### Few sounds of staff–patient engagement

While silence was descriptive of this environment, there was some engagement. Occasionally, a nurse would go to the mother to ask a question or fill in a note in the folder, but otherwise very little human contact was displayed between people.

*“I haven’t spent much time with the patients to really find out honestly how they are doing, how they have coped.” (Obstetric Registrar, Dr J, 29 years old)*

*“I think that from a psychosocial point of view we are failing our patients because we come and write their needs, are you okay mummy? Do you need to speak to anyone? Okay, bye. And that’s about the extent of our interaction with the mums. If they are very distressed and the nurses are like, oh, doctor, the patient is very upset, then we’ll sit with them a little bit longer. But it’s like minutes, maybe five or ten minutes.” (Medical doctor, Dr M, 28 years old)*

Only one doctor monitored the ward per day and was called when needed. The doctor was alone as she walked from room to room and asked the occasional question – only medically related questions. My observation led me to feel pervasively lonely and I imagined that this is what the mothers and the staff were feeling. No life. No vibrancy for a ward that is synonymous with life and new beginnings. Another medical doctor commented,

“*So usually it is quite stressful because 99 percent of the time we’re the only doctor, say, in the ward at the time. So it does take quite a bit of concentration and effort because you need to focus and kind of know which patients require reviews; how often they need to be reviewed if there are certain things you’re more worried about. So there’s a lot of multi-tasking, if I can say it like that.” (Obstetric Registrar, Dr J, 29 years old)*

*“I’m not a huge fan of talking about personal things, so I tend to not check in with the patients on an emotional level. I’m kind of like, I have questions that I want to ask, answer them, and then I’m walking away kind of thing.” (Intern Medical doctor, Dr G, 27 years old)*

When there is talk, it is often impersonal and lacking in empathy.

I attended ward rounds with the doctors on occasion. The rounds were conducted in English with a predominantly White staff. I noted it was “typical doctor interaction” where there was some engagement with the patient but the doctors often used medical terms that even I, as an English speaker, had to ask for clarification on. Patients were not addressed by their names but were often spoken about using their diagnosis.

*“I think I do do that, I do see them as a diagnosis. I mean, you’ve joined our ward rounds and we would be like, oh, this is a 35, para 3, and I’m like, no, it’s a person. With adults I find it easier to kind of create that barrier. But ja, I do tend to see patients as a diagnosis. I’m not good with names so I tend to not make the effort.” (Intern Medical doctor, Dr G, 27 years old)*

Some unintentional micro-aggressions were noted, such as when a young White intern doctor called an older mother “*sweetheart*” and then used a condescending tone to explain why she was still in the ward. The demographics of almost all the mothers are Xhosa speaking Black females. I noticed there was an awkwardness between the non-Xhosa staff and the patients.

“*I must be honest, here I’m more careful about what I say. Not that I have said anything there, but I’m more careful with regard to offending someone because I’m hyper-aware of the fact that I’m not from their community. So you tippy-toe around the fact that you are not from their community and you don’t want to say something that’s going to perhaps make it worse where they see you as this outsider.”* (*Community Service Medical doctor, Dr T, 26 years old*)

I wondered how the mothers felt being in their own community but not able to speak their home language to the doctors. Is the language further isolating, as when language is spoken to the mothers it is very medicalised and not in Xhosa? One morning, I entered the labour ward and the doctors were listening to a case presentation in very medicalised language that I could not understand. I felt very awkward. Many thoughts of “I don’t belong here”, “I don’t want to be here”, “I am not sure where to sit” lead me to think about these mothers entering this well designed and well-resourced hospital but feeling they had entered a foreign land and feeling alienated. The experience of one English speaking medical doctor articulated relief at not being able to engage more with patients,

*“I think the language barrier does actually benefit me in that sense, whereas I think my colleagues who can speak Xhosa and Zulu probably get a lot more of the emotional side of things because they can communicate better with the patients.” (Intern Medical doctor, Dr G, 27 years old)*

Policy and practice inclined away from positive milieu.

The hospital’s labour ward policy also had an impact on the ward milieu. For example, I attended a Morbidity and Mortality meeting regarding the labour ward. This was a dedicated hour to discuss with the obstetric team, and anyone else who is interested, what went wrong in the ward and how the staff could learn from the experience. The discussion was medical (almost like discussing a mathematical equation). Emotion or feelings were not discussed. I wonder if there is space to discuss emotions in a meeting like this. The staff were not engaged, as noted by how many played on their phones, and a doctor even fell asleep. It felt like women are not seen as more than their medical condition. They are not acknowledged as more than their symptoms. The women did not appear to be seen as mothers, aunts, friends, lovers, daughters. Is this a departmental defence to keep the staff removed from the pain of engagement? Some of the staff, however, seemed aware of the apparent issue,

*“People forget that its people and they become wrapped up in treating the disease and they are not treating the person.” (Obstetrician E, 36 years old)*

Both doctors and nurses also spoke to apparent gaps in training, specifically crossing cultural divides. While there was no actual mention of divides, the use of phrases like ‘those patients’ and ‘these patients’ in the quotes below speak to an us-and-them perspective,

*“But when we started nursing, no, there’s no training. It’s only about the policies that tell you which documents to complete. It’s not specifically related to the personal stuff and how to care for those patients or counselling.” (Professional Nurse & Midwife M, 35 years old)*

*“My feeling is that all staff should be trained on how to manage these patients emotionally and not just medically.” (Obstetrician P, 38 years old)*

*“When you study to become a doctor you get taught how to be a doctor, but unfortunately you don’t get taught how to be a human being.” (Obstetrician P, 38 years old)*

*“But when we started nursing, no, there’s no training. It’s only about the policies that tell you which documents to complete. It’s not specifically related to the personal stuff and how to care for those patients or counselling.” (Professional Nurse & Midwife M, 35 years old)*

When asking the staff about debriefing, all the staff said none was offered and that they often leave work with unprocessed feelings.

“*Maybe they don’t think it’s necessary. I guess maybe if I felt like I needed to speak to them* (referring to the consultants)*, I could, I just never thought about it.” (Intern Medical doctor, Dr B, 25 years old)*

“*And after that I felt actually traumatised and I sort of avoided going to the labour ward for a while. But you have to get back into it because there’s no other option.” (Medical doctor, Dr M, 28 years old)*

*“They don’t care much about the counselling of the staff.” (Professional Nurse & Midwife, Q, 35 years old)*

*“So generally if you were to bring that up in the meeting in the mornings, but there’s no specific debriefing session.” (Community Service Medical doctor, Dr T, 26 years old)*

“When you are here, you are too busy. So we don’t have time for those counsellings. I think it’s a lack of time. I think so.” (Professional Nurse G, 29 years old).

In a stressed environment where emotions are not allowed to be spoken about, sometimes the emotions are acted out. This is what I thought about when one of the professional nurses said to me:

*“Sometimes the doctors are hard with us. But not all the doctors, just some of the doctors. And also some of my colleagues, those who are experienced in labour ward. Like I’m not that experienced in labour ward. I never worked in labour ward before I came here to this labour ward.” (Professional Nurse & Midwife F, 47 years old)*

#### High turnover of staff and patients

KH is a district training hospital which means that there are numerous interns and community service health practitioners rotating through the hospital. Every quarter the hospital receives an influx of new faces. Many health practitioners have expressed that the hospital is but a stepping stone to further specialising or gaining more experience before moving to a private facility. This results in people leaving and posts not being filled for many months. Locum staff are heavily depended upon.

*“The doctors … it’s also sometimes a challenge because there are new doctors maybe every second or third month, so there is that change. When we get used to these doctors, then we need to teach the other doctors who are new from the school to fit them in.” (Professional Nurse & Midwife B, 37 years old) “Working in Khayelitsha, it’s beautiful, but there are a lot of challenges and workload than in other areas that I was exposed to. The first challenge that we have here, it’s short staffed.” (Professional Nurse & Midwife A, 32 years old)*

The labour ward has not only a high turnover of staff but also a high patient load.

*“Working in the labour ward is nice, but it is also challenging with the shortage of staff and being busy with … like Khayelitsha has a lot of patients and this hospital sometimes is not enough to cater for everyone. So that’s the challenge we have. Because sometimes we’ll be full and the patients … there won’t be beds … so we have to struggle and we have to keep some of the patients in chairs. That’s another challenge that we’re experiencing. And when you ask the patient to sit in the chair, it’s not nice, and they don’t take it very nice. And sometimes now you feel guilty because they just gave birth and they have to sit on a chair, which is not nice if you take it to yourself.” (Professional Nurse & Midwife, Q, 35 years old)*

### The world the mother brings with her

The impoverished setting of KH, and the predominantly Xhosa patient profile brings with it a stark contrast to labour wards in more privileged settings. No visitors sat with the expectant mothers as they waited for the new life. This added further silence to the deathly quiet ward. Was this not supposed to be a life changing, wonderful experience for these expectant mothers, but instead they lay quietly in their beds alone. Phones seemed to be the only other connection to the outside world for the mothers which they were told to put on silent.

When I interviewed a senior professional nurse about the visitors, she told me:

“*Visiting times are 3pm to 4pm after the woman has delivered but no visitors before unless the woman is in active labour (4cms or more dilated), then we call someone in her family. The women usually do not want someone to come because they do not want people looking down there* (she motioned towards her vagina). *We love someone supporting them.*” *(Professional Nurse G, 29 years old)*

Birthing companions were rarely seen despite the literature claiming that a birthing companion reduces the need for medical interventions, including medicated births, and improves both maternal and neonatal outcomes [38].

*“You know, some of them, culturally, we as Black people, it’s few of them that would want someone to be with them when they are in labour. And even when they go to the clinics they are being told that when you are in labour someone can come, and they can sign that companion form. There’s a form that most of the clinics give them. They can come, they know that, but we as Black people, like the boyfriends, they don’t want to see someone who is giving birth. Because even when they come to drop the stuff for them, if you ask them, boetie, can you stay? They say, no, no, no, I don’t want to be in, I don’t want to. So sometimes some of them chase their partners away and they’ll say, oh, go, you are the one who is giving me pain, I don’t want to see you.” (Professional Nurse & Midwife M, 35 years old)*

While no single cause would explain the silent milieu in the ward, the combination of these themes compounded into a significant distortion of an ideal labour ward as discussed below.

## Discussion

A silent milieu characterises the KH labour ward. In contrast to the wards ‘new life’ symbolic nature, this silence was unsettling as a participant observer. From our numerous observations and discussions with staff and patients, it is clear that no one is being overtly mistreated and all are medically well attended to. Could we categorise this silence as obstetric or gentle violence according to Chadwick’s [4, 5] definitions? While the definition of gentle violence is fairly new and inclusive [4], we believe that a silent labour ward milieu as experienced in KH should be included in the definition for a number of reasons.

First, the hospital itself has some systemic orientations that lead to silence. Meetings are strictly medical and the patients’ feelings are not factored into overall patient care. The result of this is a systemic neglect of emotional pain and a minimisation of visits and discussion with patients. The multiple patients in the labour ward also mean that visitors are not allowed into the space for privacy related reasons. The lack of visitors adds to the silence. General visiting hours (15 h00-16 h00) are also narrow and inconvenient for anyone who works a full day.

Second, the hospital staff structure and the staff themselves contribute to the silent milieu. Understaffing means that the staff on duty have little time to stop and connect with patients. They move quickly between beds just checking on them medically before moving on. Lack of training amplifies this as the doctors feel helpless to cross the cultural and socio-economic divide. While acknowledging these problems, there seems little incentive to actually change as most medical doctors are on short rotations and will move on from the labour ward within weeks. Silence can, therefore, be seen as a defence as the less you talk to someone, the less you build a relationship and you are thus able to depersonalise the individual. Menzies Lyth [13] addressed how staff defended against anxiety with the employment of defences such as splitting. She stated that splitting is employed as a defence by breaking up the work in a way that limits contact with patients. The same applies to referring to patients as diagnoses. Their personhood is stripped away. No debriefing for the staff again reinforces the denial of feelings and inevitably trains the health care providers not to become involved in patients’ emotional affairs. The staff’s feeling too are not acknowledge and they are left unseen and silenced.

Finally, the patients themselves passively contribute to the silence in a number of ways. The patients bring with them a world very unfamiliar to many of the staff as most speak Xhosa as a first language. Some of the mainly English and Afrikaans speaking doctors admitted that this language barrier was a relief as they did not know how to engage emotionally. T Although we saw no physical abuse, silence (actual and symbolic) in such a place may be interpreted as evidence of neglect, aloneness and a submissive compliance from patients. Silence can be a form of neglect as it leaves the women not cared for and not seen. Chadwick [4] specifically addresses this form of gentle violence where women accept that they are patients and comply with what they think is expected of them. Only women who have had a complication in a previous pregnancy or in this pregnancy are brought to the hospital to deliver, otherwise they deliver in the community health clinic. Are these mothers more susceptible to defensive neglect because they fear the pending outcome of the delivery which could result in trauma to themselves or the new life inside of them? Maybe it is easier to be alone and quietly deal with the prognosis like one in a medical ward trying to deal with a physical ailment that takes time, medication and rest. Maybe the women and the staff do not feel that this time is a rejoicing time as the outcome is uncertain.

Does the fact that they are in a hospital lead to furthering the silence – as a sign of powerlessness – as they have accepted their role as a patient? The women are made to enter a hospital setting, get into a hospital gown and get into a bed and assume the sick role which is normalised and accepted. With the sick role assumed, the health care practitioners do become the experts, resulting in the women feeling inferior. The medical language is communicated in a language that is not the mothers’ mother tongue, further establishing unequal power dynamics. This is congruent with Morales et al.’s [2] findings that obstetric violence occurs in a social environment favouring the development of power relationships between patients and health care practitioners. Morales et al. [2] stated that violence can be expressed through equitable access to health care and that in a poorer community they must invariably accept what they are given.

## Conclusion

We agree with Morales et al. [2] who stated, “*neither medicine nor health care staff are violent by nature*” (p. 7). Most enter the health world wanting to help. In Khayelitsha we saw this to be true. Staff provide excellent medical care and will engage when they need to. Patients’ basic needs such us food, blankets and pillows are attended to. The staff will answer all questions in a non-hostile manner and even assist the patients to the bathrooms or help with making phone calls to family members. However, in a stressed environment where most of the birthing mothers are traumatised, both staff and birthing women appear to keep quiet to defend against the anxiety that is aroused in them. The women willingly submit themselves to the supposed experts and the supposed experts quietly perform their tasks. Silence is protective for both the staff and the mothers. No one gets emotionally involved and therefore no one hurts. There are no clear perpetrators as the organisational system is built on a community that is powerless and lacks agency. Both staff and patients are victims and both collude with the defensive neglect of silence.

We do think staff need to be held accountable for their actions, as addressed by Chadwick [5], but we think they need to be supported as much as their patients need to be. In order to create a favourable milieu in the ward, all parties need to be thought about and held in mind.

Silence is always difficult to interpret because it is silence. Through this paper we have suggested various interpretations of why there is silence. One of the doctors said the following:

*“I think the nurses and the doctors here are really doing the best that they can. I think our patients are getting excellent care from a medical point of view. But our population needs so much attention, and they don’t need attention from the doctor on call for one day. This population needs attention.” (Medical doctor, Dr M, 28 years old)*

In this the participant describes not a lack of care, but an appreciation of the extent of the need and the deprivation of this community. Silence can therefore been seen as possibly an act of gentle violence, but we see it more as a way a system defends against what it knows it cannot provide.

## Data Availability

Not applicable.

## Abbreviations

FANI: Free Association Narrative Interview
KH: Khayelitsha Hospital
